# Genome Analysis and In Vitro Assay of Probiotic Properties of *Bacillus paranthracis* YC03 with Urate-Lowering Potential

**DOI:** 10.3390/microorganisms13040798

**Published:** 2025-03-31

**Authors:** Xiaoyu Cao, Yu Zhang, Qianqian Xu, Hai Yan

**Affiliations:** School of Chemistry and Biological Engineering, University of Science and Technology Beijing, Beijing 100083, China; d202210452@xs.ustb.edu.cn (X.C.);

**Keywords:** *Bacillus paranthracis* YC03, uric acid, biodegradation, genome analysis, probiotic properties

## Abstract

Hyperuricemia is a metabolic disorder owing to the underexcretion and/or overproduction of uric acid (UA). Recent studies have shown that probiotics have many potential applications as functional foods to ameliorate hyperuricemia. In this study, we have successfully isolated *Bacillus paranthracis* YC03 with urate-lowering potential from Jiangshui. The in vitro results indicated that YC03 exhibited strong biodegradation capacities toward UA and its precursors (inosine and guanosine). Meanwhile, the cell-free extracts of YC03 were also found to catalyze and remove inosine and guanosine. To further explore the application potential of this strain in developing functional foods, we evaluated its probiotic properties through in vitro assays and whole genome analysis. *B*. *paranthracis* YC03 has excellent abilities, with tolerance to acid and bile salt and good adhesion. In addition, hemolytic assays, along with antibiotic resistance and biogenic amine production tests, have also preliminarily confirmed the safety of using YC03 in food. We have also annotated the key enzyme genes, including *auaG*, *hpt*, *rih*, *punA* and *deoD*, which are involved in the biodegradation of UA and nucleosides. The results of nucleoside biodegradation product detection will be valuable for exploring the metabolic pathway for biodegrading nucleosides with YC03. These findings provide meaningful insights for the development of functional foods to improve hyperuricemia using *B*. *paranthracis* YC03.

## 1. Introduction

Hyperuricemia is a common metabolic disorder, which is defined by increased serum uric acid (UA) above the limit of monosodium urate (6.8 mg/dL) [1]. Hyperuricemia often results from disorders of purine metabolism and/or impaired UA excretion [2]. UA is the end product of purine metabolism in humans, and chronic elevation above the saturation point of monosodium urate crystals could lead to gout [3]. Meanwhile, hyperuricemia can cause inflammation or oxidative stress, which increase the risk of developing chronic kidney disease [4], cardiovascular disease [5] and type 2 diabetes [6]. Recently, the prevalence of hyperuricemia has increased significantly in the world; in mainland China, the prevalence is 22.7% in males and 11.0% in females [7], and in U.S., it is 20.2% in males and 20.0% in females [8].

Around 80% of UA is endogenous purine from damaged and dead cells, and the remaining 20% is an exogenous pool of purine that varies significantly with dietary intake [9]. UA cannot be degraded to allantoin due to the absence of uricase in humans [10]. Thus, the excretion of UA primarily occurs via two pathways: kidney excretion and intestinal excretion [11]. Currently, the main way to improve hyperuricemia is through chemical drugs to inhibit UA synthesis or increase its excretion, but also through dietary interventions to reduce purine intake [12]. However, the long-term use of chemical drugs can cause hepatorenal dysfunction and allergic reactions [13]. Dietary intervention is less effective than chemical drugs because it is hard for patients to adhere for a long time. Hence, it is necessary to develop more effective methods for the amelioration of hyperuricemia and gout.

In recent years, the amelioration of hyperuricemia using probiotics has become a research hotspot. Probiotics could reduce UA production by degrading its precursors, inhibiting the activity of enzymes related to UA synthesis and regulating the expression of UA transporters [14]. Inosine and guanosine are the primary precursors of UA [15]. Several probiotic strains showed a urate-lowering potential by biodegrading inosine and guanosine into hypoxanthine and guanine, which reduced the absorption of nucleosides by intestinal epithelial cells. However, there were rarely probiotics that exhibited this biodegradation capacity toward UA.

“Jiangshui” is a traditional fermented food in Northwestern China, which mainly uses lactic acid bacteria, acetic acid bacteria and yeast in the fermentation process [16]. There was a survey of 180 citizens in Lanzhou that indicated that there was a negative correlation between the intake of Jiangshui and the morbidity of gout [17]. *Limosilactobacillus fermentum* JL-3 and GR-3, which were isolated from Jiangshui, exhibited the ability to degrade UA [17,18]. Thus, the aim of the present study is to isolate bacterial strains from Jiangshui with UA and/or nucleoside biodegradation ability. We have successfully isolated *Bacillus paranthracis* YC03 from Jiangshui, a facultative anaerobic bacterium, which could biodegrade both UA and nucleosides. Additionally, we have preliminarily assessed the probiotic properties and safety of *B. paranthracis* YC03 through combining a whole genome analysis and in vitro assays. This study holds significant importance in the development of functional foods for ameliorating hyperuricemia and gout.

## 2. Materials and Methods

### 2.1. Chemicals and Samples

UA, inosine, guanosine, hypoxanthine and guanine (above 99% purity) were purchased from Aladdin Chemical Co. (Shanghai, China), and all other reagents used for experiments were analytical grade. The bacterial strain used in this study was isolated from traditional fermented Jiangshui samples, purchased from market (Pingliang, China). All of the experiments above were performed at 37 °C with shaking at 200 rpm.

### 2.2. Isolation of the Bacterial Strain with Uric Acid Biodegrading Ability

From the Jiangshui samples, the strain YC03 was isolated in an UA medium (500 mg/L NH_4_Cl, 500 mg/L Na_2_HPO_4_, 50 mg/L KH_2_PO_4_, 100 mg/L MgSO_4_, 10 mg/L CaCl_2_, 10 mg/L vitamin B complex tablets, 2000 mg/L UA, 0.1 mL trace element solution and 1.8–2.0% agar, initial pH 7.0) [17]. Strain YC03 was transferred to a new liquid medium with UA as the sole carbon and energy source. The YC03 cultures obtained were continuously diluted and then streaked on new LB agar plates to acquire a single colony. The YC03 monoclonal colony activated in LB medium was then stored in 50% glycerol at −20 °C.

### 2.3. Determination of Uric Acid and Nucleoside Biodegradation Ability of YC03

To evaluate its biodegradation ability for UA and nucleosides, YC03 was activated in LB medium and transferred to the UA biodegradation medium (except the initial UA of 2000 mg/L, which changed to 500 mg/L, the other ingredients stayed the same as the isolation medium) and nucleoside biodegradation mediums (replaced 2000 mg/L of UA with 1000 mg/L of inosine or guanosine). Subsequent biodegradation experiments with YC03 were performed for 48 h. The optical density (OD600) was determined using the manufacturer (INESA, Shanghai, China) to represent the bacterial growth and the UA or nucleoside concentration was determined by high performance liquid chromatography (HPLC, Shimadzu LC-20AT, Tokyo, Japan). Non-inoculated medium served as a control.

### 2.4. Genomic DNA Extraction, Sequencing, Assembly and CDS Prediction

The strain YC03 was inoculated in LB medium and incubated for 48 h. The high-quality genomic DNA of YC03 was extracted using MagPure Bacterial DNA Kit (D6361-02, Magen, Shanghai, China). DNA concentration was determined via Qubit4.0 (Thermo, Q33226, Waltham, MA, USA). DNA integrity was assessed by 1% agarose gel electrophoresis.

The whole genome DNA was randomly fragmented to an average size of 200–400 bp. The selected fragments were acquired through end-repair, 3′ adenylation, adapter ligation and PCR amplification. After purification with the magnetic beads, the library was qualified by the Qubit 4.0 fluorometer and the length of library was assessed by 2% agarose gel electrophoresis. The qualified libraries were sequenced on the Illumina NovaSeq 6000 platform at Sangon Biotech (Shanghai, China).

After sequencing, raw reads were filtered via Trimmomatic v0.36 by removing adaptors and low-quality reads, then clean reads were obtained. Genome assembly was done using SPAdes v3.15 and the Gapfiller v1.11 was used for filling gaps, which was selected for assembly due to its robust performance in handling short-read sequencing data, particularly for bacterial genomes. Gene predictions and annotations were generated using the Prokka v1.10 and NCBI database. The functional annotation of genes was mainly based on protein coding genes using the NR, GO, COG and KEGG database.

### 2.5. Identification of YC03

YC03 was identified by a combination of 16S rDNA sequencing analysis and calculation of average nucleotide identity (ANI) with genomic sequences. ANI was calculated using OAT, and a heatmap was drawn based on the results.

This Whole Genome Shotgun project has been deposited at GenBank under the accession JAWXDP000000000.

### 2.6. Determination of Nucleosides Biodegradation Ability of YC03’s Cell-Free Extracts

The strain YC03 was inoculated in 25 mL LB medium, and then the cultures of YC03 were centrifuged at 14,000 rpm for 15 min. The YC03 cells were washed three times, and resuspended with phosphate-buffered saline (PBS, pH 7.4). After that, the cells were sonicated at 4 °C with an output power of 360 W for 25 min by an ultrasonic homogenizer (JY92-IIDN, SCIENTZ, Ningbo, China). The supernatant, as cell-free extract (CE), was obtained by centrifugation at 14,000 rpm for 15 min at 4 °C, and was then used to catalyze the bioreaction of nucleosides (the initial inosine or guanosine concentration was around 430 mg/L). The concentration of protein was determined with BCA method [19]. The samples were taken at different times to measure nucleoside concentration by HPLC.

### 2.7. Analysis of Uric Acid, Inosine and Guanosine and Biodegradation Products by HPLC

HPLC was used to determine the concentrations of UA, inosine, guanosine and their metabolites followed an improved method [20]. The external standard method was then used to determine the retention time of UA, inosine, guanosine, hypoxanthine or guanine to generate a quantitative standard curve. All experiments were conducted in triplicates and were subjected to statistical analysis.

### 2.8. In Vitro Probiotic Properties

#### 2.8.1. Evaluation of the Acid and Bile Salt Tolerance

To determine the acid tolerance, *B. paranthracis* YC03 was incubated for 48 h and transferred in sterilized saline solution with varying pH (1–5) values at 2% (*v*/*v*), and cultured for 3 h. Bile salt tolerance was determined by inoculating 2% (*v*/*v*) of 48-h-old culture in sterilized saline solution containing different concentrations of bile salts (0.1, 0.2, 0.3, 1 and 2%) and incubated for 5 h [21]. The viable cell counting was performed on LB agar plates using the dilution spread plate method, with each experiment repeated three times, and the average value was calculated. A normal saline group was used as the control to calculate the survival rate.

#### 2.8.2. Auto-/Co-Aggregation Assay

The auto-/co-aggregation assay was performed as in previous research [22]. Briefly, *B. paranthracis* YC03 was incubated for 18 h and harvested by centrifugation (12,000 rpm, 10 min). The cells were washed twice with PBS (pH 7.4) and resuspended at 10^8^ CFU/mL in PBS. The cell suspensions were incubated for 5 h at 37 °C. Subsequently, aliquots were taken from the sample at 0 and 5 h, and the absorbance was measured at OD600.

*B. paranthracis* YC03 and two common pathogenic bacteria (*Staphylococcus aureus* and *Escherichia coli*) were prepared based on the auto-aggregation assay described earlier. Equal volumes of YC03 (2 mL) and pathogenic strains (2 mL) were mixed and incubated for 5 h at 37 °C. The absorbance was measured at OD600 at 0 and 5 h of incubation.

The auto-/co-aggregation percentage was calculated using the following formula: auto-/co-aggregation (%) = [1 − (At/A0)] × 100, where At represented the absorbance at time t = 5 h and A0 the absorbance at t = 0 h.

#### 2.8.3. Cell Surface Hydrophobicity

*B. paranthracis* YC03 cells were harvested and washed twice with PBS, resuspended at 10^8^ CFU/mL in PBS, and the OD600 (A0) was determined. Following this, 2 mL of n-hexadecane and ethyl acetate was added to 2 mL of cell suspension and vortexed for 2 min [21,23]. The suspension was incubated at room temperature to allow phase separation. The aqueous phase was removed and its absorbance at 600 nm (A1) was read. The cell surface hydrophobicity was calculated as (%) = [1 − (A1/A0)] × 100.

### 2.9. Safety Assessment

#### 2.9.1. Antibiotic Resistance Test

The antibiotic resistance of *B. paranthracis* YC03 was assessed using the macrodilution broth method according to the guidelines of the Clinical and Laboratory Standards Institute (CLSI) [21,24]. Eight antibiotics were used and their concentrations ranges are as follows: chloramphenicol (0.12–64 mg/L), clindamycin (0.12–64 mg/L), tetracycline (0.12–128 mg/L), gentamicin (0.03–64 mg/L), kanamycin (0.12–64 mg/L), vancomycin (0.12–64 mg/L), erythromycin (0.12–64 mg/L) and ampicillin (1–128 mg/L). A total of 100 μL cell suspension (10^7^ CFU/mL) was transferred to the MH mediums and incubated at 37 °C for 24 h. The minimum inhibitory concentration (MIC) values were determined by visual observation of the turbidity. The susceptibility or resistance of YC03 to specific antibiotics was determined based on the MIC thresholds established by the European Food Safety Authority (EFSA) for *Bacillus* strains [25].

#### 2.9.2. Hemolysis Assay

*B. paranthracis* YC03 was plated on Colombian CNA blood agar plates. After incubation at 37 °C for 24 h, signs of β-hemolysis (complete hemolysis), α-hemolysis (incomplete hemolysis) or γ-hemolysis (non-hemolysis) were observed [21].

#### 2.9.3. Biogenic Amine Production Assay

Production of biogenic amines was performed by inoculating 100 μL of YC03 cell suspension into the amino acid decarboxylase control broth, lysine decarboxylase broth, ornithine decarboxylase broth and L-arginine decarboxylase broth [26]. After that, 300 μL of sterilized liquid paraffin was added into each tube and then tubes were incubated at 37 °C for 24 h. The appearance of purple in the broth indicated that the corresponding biogenic amine was produced.

### 2.10. Statistics Analyses

The data were calculated, analyzed and plotted by Microsoft Excel and Origin 2021. The biodegradation ratio was calculated according to the following formula: (C0 − Ct)/C0 × 100%, where C0 is initial concentration of UA, inosine or guanosine (mg/L) and Ct is residual concentration of UA, inosine or guanosine in the sample (mg/L).

## 3. Results and Discussion

### 3.1. Isolation and Identification of Uric Acid Biodegrading Strain

**Figure 1:**
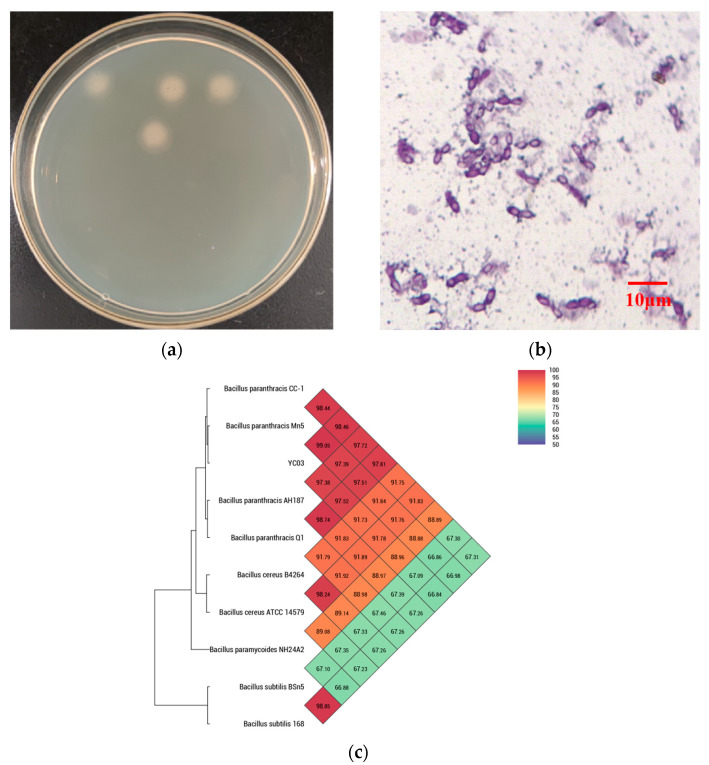
Isolating uric acid biodegrading strain YC03. Colonies were grown on LB agar plate (**a**); morphology (1000×) under the microscope (**b**); and heat map of average nucleotide identity (**c**).

The monoclonal colonies of strain YC03 were grown on a LB agar plate (Figure 1a), which showed the off-white color, as well as the circular and non-translucent morphology of YC03 colonies. The YC03 strain cells are Gram-stain-positive, facultatively anaerobic, nonmotile and had a central elliptical endospore, which was observed under a light microscope with 1000× magnification (Figure 1b).

16S rDNA sequencing analysis confirmed that YC03 belongs to the genus *Bacillus*. Microscopy also showed that YC07 had a typical morphology for *Bacillus* sp. The genome of YC03 was 97.84% identical by ANI to the type genome of *B. paranthracis* (Figure 1c). *B. paranthracis* is a novel species of the *B. cereus* group [27], and there have not been many relevant studies. In previous research, *B. paranthracis* strain DB-4 was also isolated from fermented foods, and a draft genome analysis showed that *B. paranthracis* strain DB-4 may remove reactive oxygen species in the fermentation process [28]. *B. paranthracis* strain ICIS-279 [29] and strain MHSD3 [21] were isolated from the human intestine and sterilized leaves of a medicinal plant, respectively. Both *B. paranthracis* ICIS-279 and MHSD3 may contribute to probiotic development, and the probiotic features of them were explored using genomic analysis and in vitro experiments. However, to the best knowledge of the authors, there is no previous research regarding UA biodegradation by *B. paranthracis*. *B. paranthracis* YD01 isolated from healthy individual feces only exhibited the biodegradation capacity toward nucleosides [20].

### 3.2. Biodegradation of Uric Acid and Nucleoside by B. paranthracis YC03

**Figure 2:**
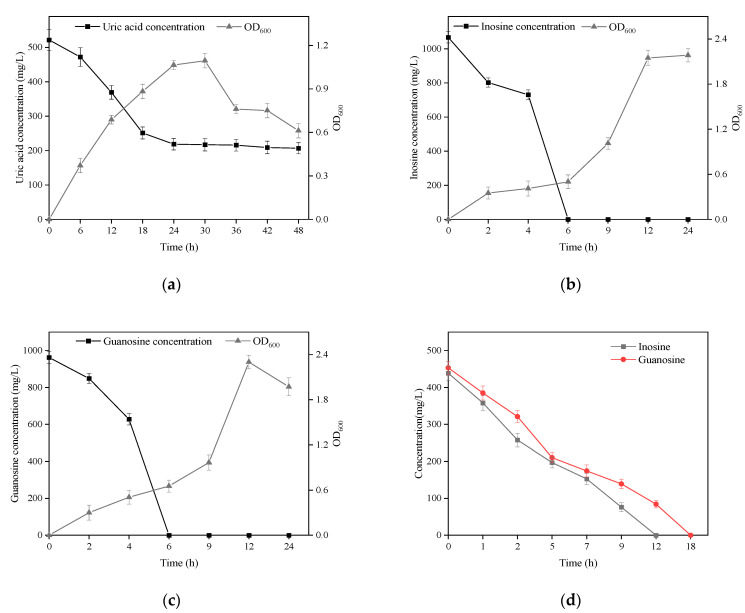
Growth and biodegradation curves of YC03 in mediums using uric acid (**a**), inosine (**b**) and guanosine (**c**) as the sole carbon and energy source. Biodegradation curves of inosine and guanosine in PBS by YC03’s CEs (**d**).

*B. paranthracis* YC03 with UA biodegradation capabilities was isolated from traditional fermented Jiangshui and cultured in a medium containing UA as the sole carbon and energy source. The strain YC03 could survive when the OD600 reached above 1.1, and the initial UA of 520 mg/L was removed to give a concentration of 205 mg/L, which showed a biodegradation ratio above 60.5% within the 48 h of the incubation period (Figure 2a). We found that the biodegradation of UA was mainly within 24 h, after which the concentration of UA did not change significantly. Later, to analyze the inosine and guanosine biodegrading ability of YC03, it was added to the medium containing inosine or guanosine as the sole carbon and nitrogen source for cultivation, respectively. The results indicated that YC03 could completely biodegrade initial inosine or guanosine concentrations of 1000 mg/L within 6 h, showing a 100% biodegradation ratio, and the OD600 could reach above 2.3 or 2.2, respectively (Figure 2b,c). Furthermore, the CE of YC03 containing a protein concentration of 653 mg/L could completely remove 423 mg/L of guanosine within 18 h and 438 mg/L of inosine within 12 h (Figure 2d), which showed that the biodegradation ability for inosine was better than that of guanosine. These results indicate that YC03 has a better ability to biodegrade nucleosides than *B. paranthracis* YD01, which exhibited biodegradation capacities towards inosine or guanosine of only 50 mg/L within 12 h [20]. Alternatively, the HPLC results revealed absorption peaks of inosine or guanosine and their biodegradation products during biodegrading process.

Recently, studies showed that several probiotics could assimilate and degrade nucleosides (inosine and guanosine) to lower the levels of metabolites, such as ribose and base, and then UA synthesis is slowed with the reduction in these intermediates. *Lactobacillus* is the main category of probiotics that can reduce the levels of serum UA through multiple pathways, such as, *L. fermentum* 9-4 [30], *L. brevis* MJM60390 [31] and *L. gasseri* PA-3 [32]. Thus, it can be seen that these potential strategies to ameliorate hyperuricemia aim to reduce UA synthesis through biodegrading nucleosides. In in vivo experiments, probiotics also have alleviative effects on hyperuricemia via anti-inflammation and gut microbiota homeostasis [33]. Next, the UA-lowering ability of *B. paranthracis* YC03 in vivo will be the focus of our research, and then we will discuss possible therapeutic pathways. Among the available studies, probiotics as food supplements have potential benefits in the amelioration of hyperuricemia and gout, and were reported to decrease UA production through degrading purines and inhibiting xanthine oxidase activity, as well as increasing UA excretion by promoting the expression of UA transporters [14]. However, there are few probiotics that can directly metabolize UA; *B. paranthracis* YC03 can not only reduce UA biosynthesis by metabolizing purines to compounds other than UA, but can also biodegrade UA to the more soluble compound allantoin.

### 3.3. Nucleoside Biodegradation Products Identification

**Figure 3:**
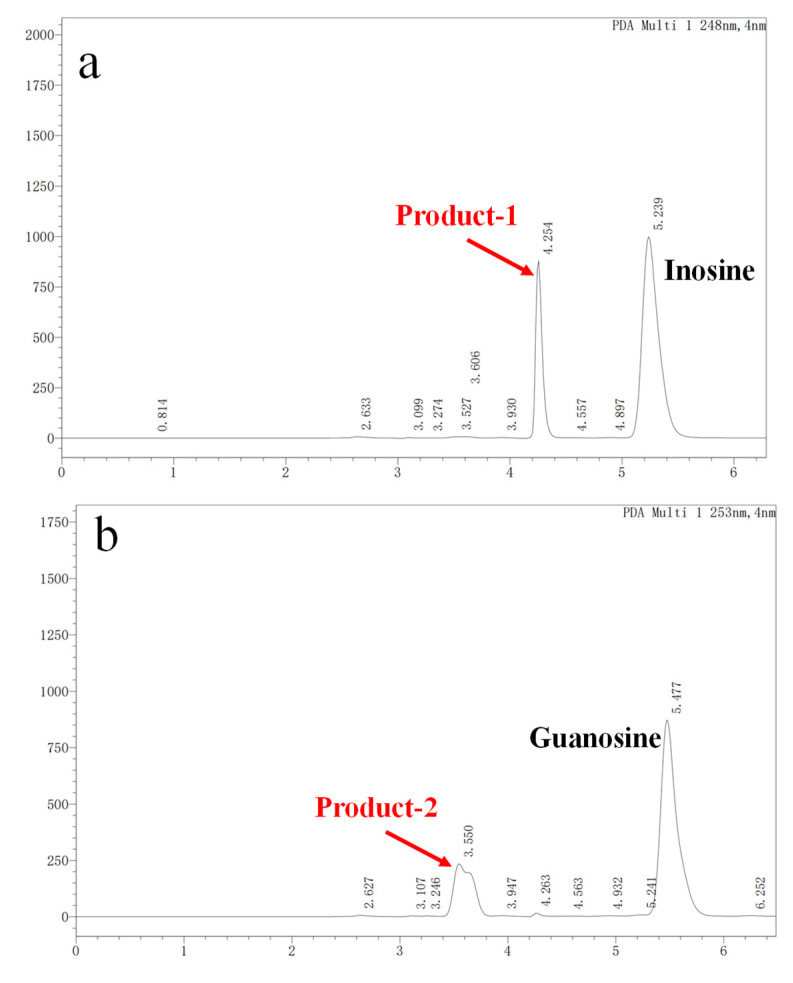

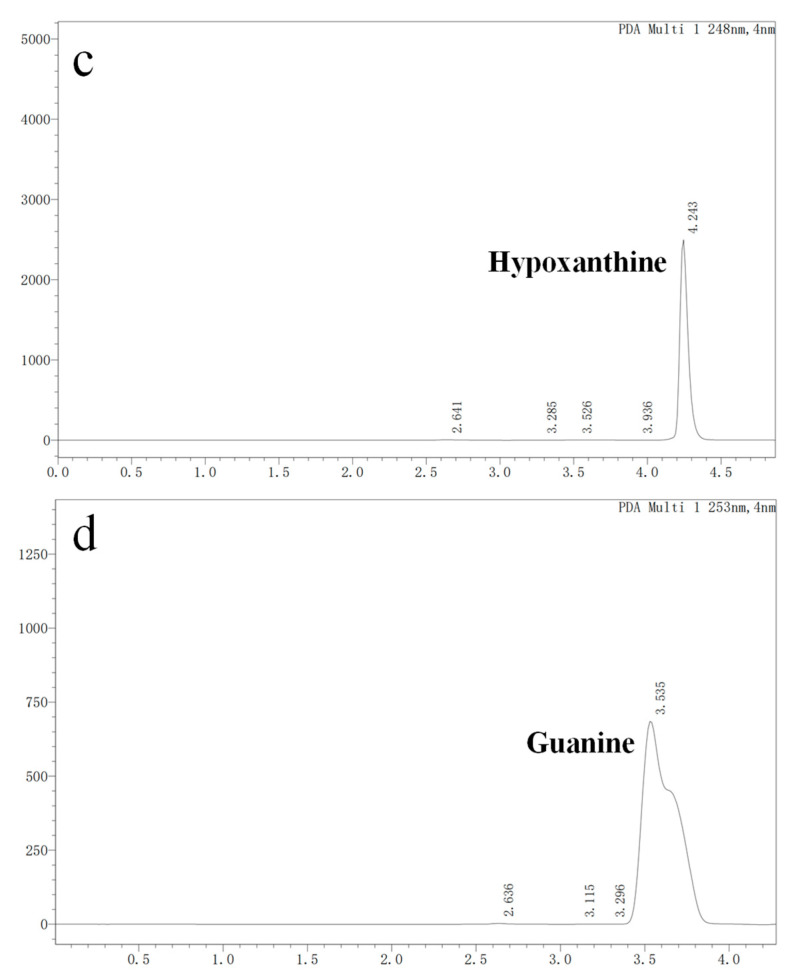
Inosine and guanosine biodegradation products of YC03 detected by HPLC. Inosine and its biodegradation product (**a**); guanosine and its biodegradation product (**b**); detection peak of hypoxanthine standard (**c**); and detection peak of guanine standard (**d**).

In vitro degradation experiments of inosine and guanosine with LAB had been performed, and the results showed that the compounds detected in inosine and guanosine assimilation were inosine, guanosine, xanthine, hypoxanthine, guanine and UA [34]. In this study, the HPLC result (sampling at 4 h) showed that the absorption peaks of inosine and that of its biodegradation product were at 5.239 and 4.254 (Figure 3a), which was consistent with the standard of the hypoxanthine (Figure 3c) peak. Another HPLC result revealed that the absorption peaks of guanosine and its biodegradation product were at 5.477 and 3.550 (Figure 3b), which was consistent with the standard of the guanine (Figure 3d) peak. However, no further products were found in the HPLC results between 6 h and 24 h. As the results showed that YC03 dramatically decreased the inosine and guanosine, hypoxanthine and guanine were the two main components of the metabolites, and almost 100% of the consumed inosine and guanosine were very likely converted to hypoxanthine and guanine, respectively. These results are coincident with former reports, as its biodegradation ratio was found to be more significant compared with *Lactiplantibacillus plantarum* [35].

### 3.4. Overview of Genome Analysis

The draft genome sequence of strain YC03 was 5,533,921 bp, with an average GC content of 35.22%. The reads were assembled into 58 scaffolds with an N50 of 300,358 bp. A total of 5683 CDS genes, 96 tRNA genes, 10 rRNA genes and 1 ncRNA gene were predicted. *B. paranthracis* YC03 had a similar genomic GC content and genome size compared with other reported *Bacillus* strains [21].

The results of the genome annotation revealed that 5671 genes of YC03 were annotated in the Non-Redundant Protein Database (NR), 3589 genes were annotated in the Pfam Database, 3571 genes were annotated in the Clusters of Orthologous Groups of Proteins Database (COG), 2822 genes were annotated in the Gene Ontology Database (GO) and 2206 genes were annotated in the Kyoto Encyclopedia of Genes and Genomes Database (KEGG).

**Figure 4:**
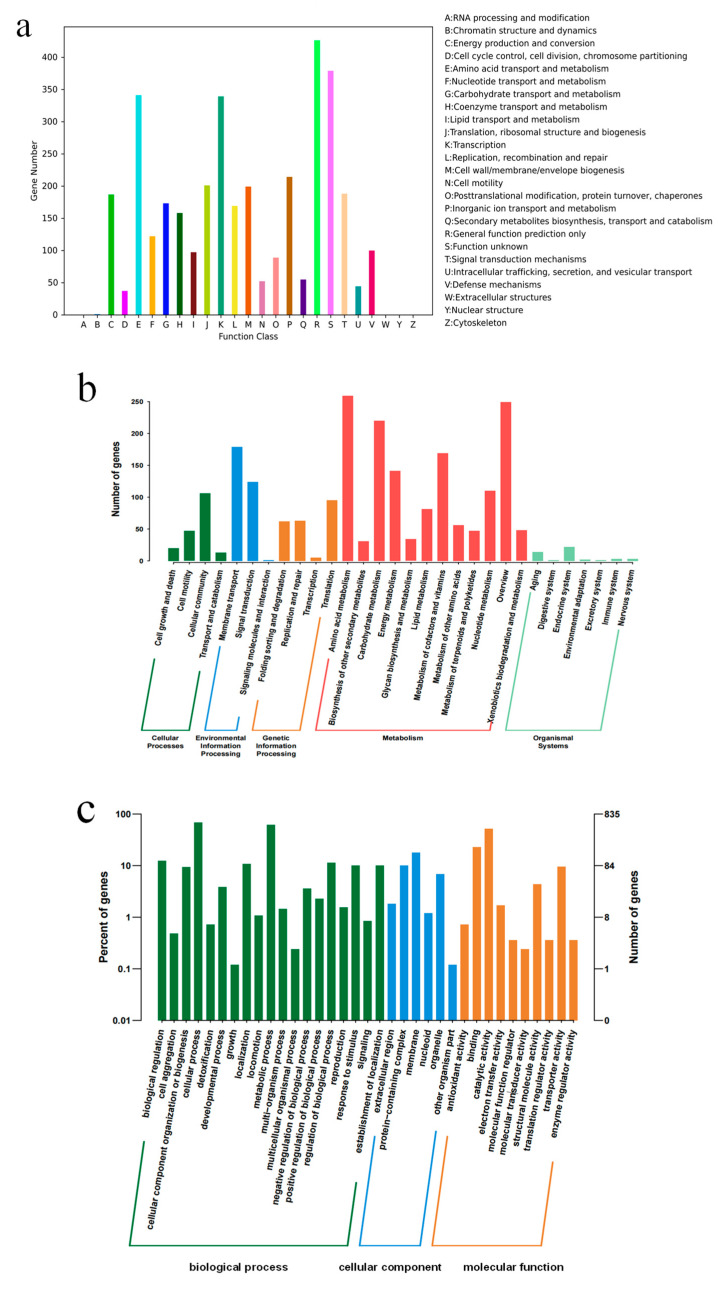
COG (**a**), KEEG (**b**) and GO (**c**) annotation classification of *B. paranthracis* YC03.

The genome annotation results of YC03 revealed that 3571 genes were categorized into 26 different categories of the COG (Figure 4a). Notably, 341 genes were associated with amino acid transport and metabolism (E), 122 genes with nucleotide transport and metabolism (F), 173 genes with carbohydrate transport and metabolism (G), 158 genes with coenzyme transport and metabolism (H) and 97 genes with lipid transport and metabolism (I). It is worth mentioning that the gene ctg00037-05835, encoding for the pyridoxine biosynthesis enzyme, was annotated based on the COG analysis. Pyridoxine (vitamin B6) is a component of some coenzymes in the human body, and plays a crucial role in many cellular metabolic processes, especially with amino acid metabolism. Additionally, 2206 genes were annotated in the KEGG database (Figure 4b). There are 12 categories in the metabolism classification and 74 genes were annotated that are related to purine metabolism. Some of these enzymes are important in the de novo synthesis and salvage pathways of UA production [36]. Furthermore, 2822 genes were annotated in the GO database, with 1744 genes attributed to biological processes, 315 genes attributed to cellular components and 763 genes attributed to molecular functions (Figure 4c).

In addition, six catalase genes, including a vegetative catalase gene and a manganese catalase gene, were annotated in the NR database. They may protect lactic acid bacteria in fermented foods by eliminating reactive oxygen species. The analysis of carbohydrate-active enzymes revealed that the genome of strain YC03 contained 66 genes, 21 Glycosyltransferase (GT) genes, 14 Glycoside Hydrolase (GH) genes, 1 Polysaccharide Lyase (PL) gene, 23 Carbohydrates Esterase (CE) genes and 7 Auxiliary Activities (AAs) genes. Further analysis revealed the presence of AA7, GH13 and GH74, which are reported as major oligosaccharide-degrading enzymes, such as glucooligosaccharide oxidase, oligo-alpha-glucosidase and chitooligosaccharide oxidase. The key enzyme for cellulose biosynthesis, cellulose synthase GT2, has already been identified in the YC03 genome. GTs play a crucial role in the formation of surface structures, which could be recognized by the host immune system [37]. These results suggest the probiotic potential of YC03, especially for immune stimulation and pathogen defense.

### 3.5. Genetic Features of Probiotic Properties in YC03

First of all, the strain, being a potential probiotic species, must maintain survival in harsh conditions, remaining active after passing through the gastrointestinal environment (low gastric pH and bile). Secondly, secretion of antimicrobial components is also an important property of probiotics [21]. The *B. paranthracis* YC03 genome analysis revealed that many genes play a role in its survival in harsh conditions and its inhibition of the survival of pathogenic bacteria (Table 1). Four adhesion genes were annotated in the NR database, which encoded for the sortase-dependent surface proteins. Not only can they play a key role in processes associated with mucosal adhesion, where the gene encoding for mucus-binding protein *lspA* contributes to the adherence of bacteria to the intestinal mucosa, but they also maintain intestinal homeostasis. The “*atp*” gene is essential in maintaining a neutral pH in the bacterial cytosol. Additionally, the YC03 genome analysis also revealed genes that encode for lactate synthesis, which are associated with antimicrobial activity and acid resistance. All in all, these findings suggest that *B. paranthracis* YC03 has potential probiotic properties.

### 3.6. In Vitro Assay of Probiotic Properties of YC03

#### 3.6.1. Acid and Bile Salt Tolerance

**Figure 5:**
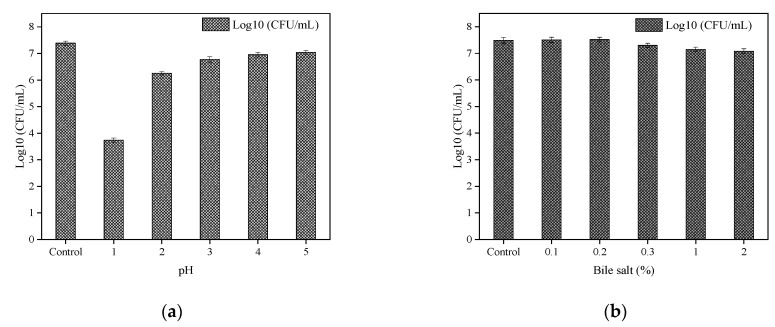
Acid (**a**) and bile salt (**b**) tolerance of YC03.

The tolerance of *B. paranthracis* YC03 to acid and bile salt is shown in Figure 5a and Figure 5b, respectively. *B. paranthracis* YC03 could survive in all bile salt concentrations, and the number of live bacteria was maintained at 10^7^ CFU/mL. Previous research recorded 50–78% survival rates for *B. paranthracis* MHSD3 in 0.05–5% bile salt concentrations [21]. However, when the pH was adjusted to 1.0, the viable bacteria decreased significantly with the extension of the incubation time, while the viable number of bacteria was still able to reach 10^3^–10^4^ CFU/mL. A similar study of *Bacillus* sp. showed a survival rate ranging from 7.14 to 96.77% at pH 1.55 and pH 4.94 [38]. As a potential probiotic candidate, the acid and bile salt tolerance tests are crucial for assessing its potential to survive the harsh conditions of the gastrointestinal tract. These results showed that strain YC03 has potential probiotic properties.

#### 3.6.2. Auto-/Co-Aggregation and Cell Surface Hydrophobicity

Adhesion to the intestinal mucosa is a crucial property of probiotic strains, as it helps prevent pathogen invasion and inflammation within the gastrointestinal tract while protecting intestinal epithelial cells [21,23]. According to Guidelines for the Evaluation of Probiotics in Food (FAO/WTO), a probiotic candidate strain must possess the ability to adhere to the mucosal surface [39]. Additionally, a good auto-aggregation capability is generally considered to be above 40% [40]. After 2 h of incubation, *Lactobacillus* strains showed the auto-aggregation capacities ranging from 21.78–66.03% [22]. *B. paranthracis* YC03 was regarded to have a good auto-aggregation capability with a value of 43.8%. In addition, ethyl acetate and n-hexadecane were used to test the cell surface hydrophobicity of *B. paranthracis* YC03. Strain YC03 showed the hydrophobicity to ethyl acetate and n-hexadecane were 13.4% and 30.2%, respectively. And YC03 was able to co-aggregate two common pathogenic strains tested. The co-aggregation was 37.8% with *E. coli* and 20.9% with *S. aureus*. Co-aggregation is a significant factor in food preservation and plays a role in pathogen elimination [41]. These results further showed that YC03 has potential probiotic properties.

### 3.7. Antibiotic Resistance

*B. paranthracis* YC03 showed resistance to tetracycline and ampicillin. Strain YC03 exhibited sensitivity to chloramphenicol, clindamycin, gentamicin, kanamycin, vancomycin and erythromycin (Table 2). We noticed that the tetracycline antibiotic gene *tet (45)* and cephalosporin antibiotic gene (*Bla1*, *BcII*) were annotated in the CARD of the YC03 genome. Typically, potential probiotic strains must be safe for use and should demonstrate minimal or no resistance to antibiotics [42]. In this context, strain YC03 qualifies as a promising probiotic candidate because it showed susceptibility to the majority of antibiotics tested in this study. However, the presence of mobile genetic elements, such as plasmids carrying resistance genes, can facilitate the spread of antibiotic resistance, thereby undermining the therapeutic efficacy of antibiotics. Therefore, a thorough evaluation of the genetic basis of YC03’s resistance to tetracycline and ampicillin is warranted to assess the risk of horizontal gene transfer.

### 3.8. Hemolysis Assay

**Figure 6:**
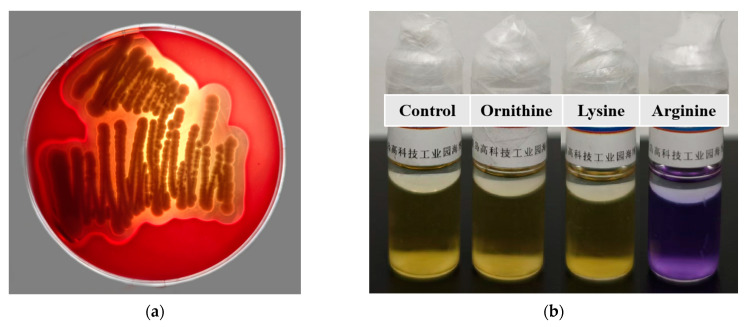
Hemolysis ability of *B. paranthracis* YC03 (**a**), which showed β-hemolysis. Determination of the ability of *B. paranthracis* YC03 to produce biogenic amines (**b**). From left to right are amino acid decarboxylase control broth, ornithine decarboxylase broth, lysine decarboxylase broth and L-arginine decarboxylase broth.

After incubation on Columbia Blood Agar Base Medium for 48 h, *B. paranthracis* YC03 exhibited β-hemolysis ability (Figure 6a). It is worth mentioning that a *putative membrane hydrolase* (*hlyIII*) was annotated in the VFDB of the YC03 genome, which encodes a hemolysin. According to a previous study, several commercially available *Bacillus* probiotic strains exhibited β-hemolysis ability [25]. Despite strain YC03 exhibiting β-hemolysis abilities, it may not necessarily pose safety concerns during oral probiotic use. Therefore, in subsequent research, we first aim to determine whether this strain poses any safety issues to mice through an acute oral toxicity test.

### 3.9. Biogenic Amine Production

The ability of *B. paranthracis* YC03 to produce biogenic amines was tested qualitatively using decarboxylase broth containing three precursor amino acids. The results showed that lysine and ornithine decarboxylase are negative, while the arginine decarboxylase result is positive (purple) (Figure 6b), which indicates that YC03 can utilize arginine decarboxylase to convert arginine into agmatine. Agmatine may be further converted into spermidine. Therefore, the test results indicate that YC03 possesses arginine decarboxylase, which suggests the potential to synthesize spermidine [43]. Spermine is a common bioamine that plays an important role in somatic cells and has no clear adverse effects on health, and demonstrates potential benefits in anti-aging, cardiovascular health and neuroprotection [44]. However, whether spermidine is actually produced may require further verification, such as through HPLC or other methods for detecting polyamines.

### 3.10. Metabolism Pathway for Uric Acid, Inosine and Guanosine Biodegradation

Previous studies mainly concentrated on purine metabolism, and only a few focus on UA biodegradation. The genes and enzymes involved in the conversion of UA, inosine and guanosine were annotated based on the NR and KEGG analysis (Table 3). However, there is no gene encoding for uricase, which could convert UA to allantoin directly. However, the genes encoding for riboflavin kinase, flavin mononucleotide (FMN) adenylyltransferase and flavin adenine dinucleotide (FAD)-dependent urate hydroxylase were annotated based on the NR analysis. This result is consistent with our previous research, which indicated that the metabolic pathway of UA biodegradation by YC03 proceeds via three steps [45]. First of all, vitamin B2 is converted to FMN by riboflavin kinase and then converted to FAD by FMN adenylyltransferase. With the assistance of FAD-dependent urate hydroxylase and hydroxyisourate hydrolase, UA can be converted to 5-hydroxyisourate (HIU), which is then broken down into 2-oxo-4hydroxy-4-carboxy-5-ureidoimidazoline (OHCU) and eventually forms (R)-allantoin spontaneously.

**Figure 7:**
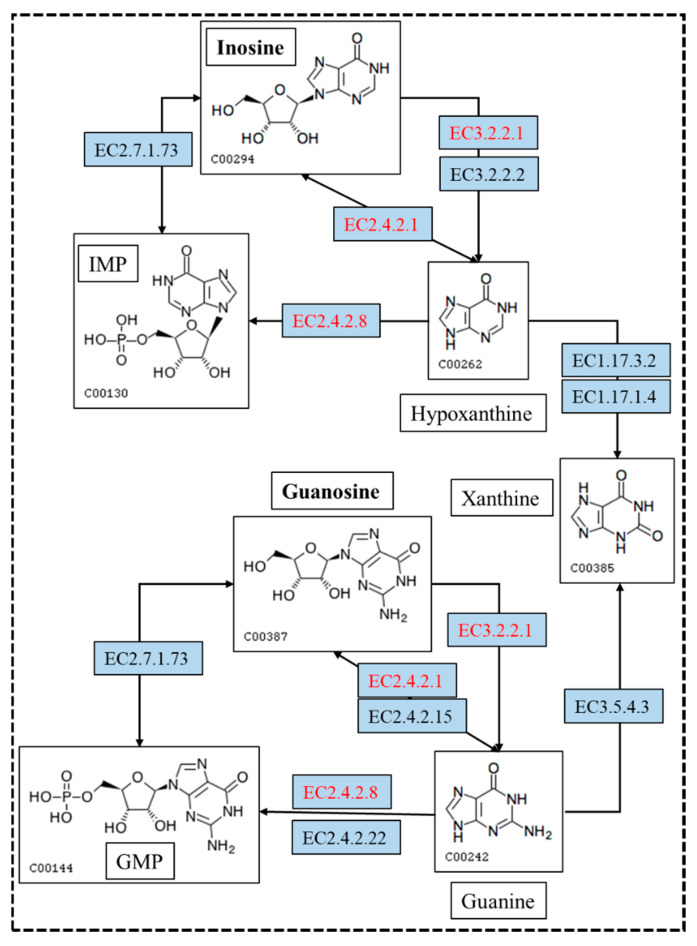
The metabolism pathway for biodegrading inosine and guanosine using *B. paranthracis* YC03. The pathways in red are annotated based on the KEGG analysis. [EC:2.4.2.8], hypoxanthine-guanine phosphoribosyltransferase; [EC:2.4.2.1], purine-nucleoside phosphorylase; and [EC:3.2.2.1], purine nucleosidase.

In the biodegradation experiments, the results showed that inosine and guanosine were very likely to be transferred to hypoxanthine and guanine, respectively. Inosine and guanosine could use purine nucleosidase, purine-nucleoside phosphorylase, inosine nucleosidase and guanosine phosphorylase to form hypoxanthine and guanine (Figure 7). Either hypoxanthine and guanine could use hypoxanthine-guanine and/or xanthine phosphoribosyltransferase to form inosine monophosphate (IMP) and guanine monophosphate (GMP), or use xanthine oxidase and guanine deaminase to form xanthine. Xanthine is an essential precursor of UA production; it is oxidized by xanthine oxidase to form the final product, UA. Importantly, the genome analysis results showed that eight genes encoding for purine nucleosidase, purine-nucleoside phosphorylase and hypoxanthine-guanine phosphoribosyltransferase were annotated based on the KEGG analysis. This result makes the biodegradation product conjecture be more credible. Despite the presence of hypoxanthine-guanine phosphoribosyltransferase, IMP or GMP were not found in the further metabolic pathway. These findings are helpful to the understanding of the molecular mechanism of YC03 biodegradation of UA and nucleosides.

## 4. Conclusions

In the present work, *B. paranthracis* YC03 from the Chinese traditional fermented food Jiangshui, exhibits strong biodegradation capacities toward both UA and its precursors. Combining in vitro probiotic assays and genomic analysis, *B. paranthracis* YC03 can be identified as a prospective probiotic candidate strain. Meanwhile, the metabolic pathways employed by YC03 in UA and nucleoside biodegradation were elucidated by gene functional annotation and HPLC detection, which will contribute new avenues for decreasing serum UA levels. These findings highlight that *B. paranthracis* YC03, as a potential probiotic, can bring profound insight into functional food in the amelioration of hyperuricemia and gout. However, several current in vitro safety tests are inadequate. It is necessary to further explore its safety through in vivo experiments, which determine whether it can be used in the food industry.

## Figures and Tables

**Table 1:** Potential genes related to different probiotic properties from YC03 genome.

Function	Gene	Product
Adhesion	*lspA*	Lipoprotein signal peptidase
	*dltD*	Protein DltD
	*dltC*	D-alanyl carrier protein
	*dltA*	D-alanine-D-alanyl carrier protein ligase
Acid stress	*atp*	ATP synthase
	*aspS*	Aspartate-tRNA (Asp/Asn) ligase
	*aspA*	Aspartate ammonia-lyase
	*recA*	Protein RecA
	*soda*	Superoxide dismutase [Mn]
	*relA*	GTP pyrophosphokinase
	*groL*	60 kDa chaperonin
	*groS*	10 kDa chaperonin
Acid stress/bile resistance	*dnaA*	Chromosomal replication initiator protein DnaA
	*dnaC*	Replicative DNA helicase
	*dnaE*	DNA polymerase III subunit alpha
	*dnaI*	Primosomal protein DnaI
	*dnaK*	Chaperone protein DnaK
	*dnaJ*	Chaperone protein DnaJ
	*dnaG*	DNA primase
	*dnaD*	DNA replication protein DnaD
Bile resistance	*nagA*	N-acetylglucosamine-6-phosphate deacetylase
	*nagB*	Glucosamine-6-phosphate deaminase
	*pyrG*	CTP synthase
	*argS*	Arginine-tRNA ligase
	*rps*	30S ribosomal protein
	*rpl*	50S ribosomal protein
Antimicrobial properties		
mdh	*alsT*	Amino-acid carrier protein AlsT
	*alsS*	Acetolactate synthase
	*alsD*	Alpha-acetolactate decarboxylase
Transcriptional regulator	*sig*	RNA polymerase
	*ctsR*	Transcriptional regulator CtsR

**Table 2:** In vitro minimum inhibitory concentrations of antibiotics for YC03. The last column includes EFSA-recommended MIC thresholds for antibiotic resistance in Bacillus strains.

Antibiotic Substance	EFSA MIC (mg/L) Resistance Threshold	MIC (mg/L)
Chloramphenicol	8	4, S
Clindamycin	4	2, S
Tetracycline	8	32, R
Gentamicin	4	0.25, S
Kanamycin	8	0.5, S
Vancomycin	4	2, S
Erythromycin	4	1, S
Ampicillin	16	64, R

MIC: Minimum Inhibitory Concentration, S: Sensitive, R: Resistant

**Table 3:** Genes and corresponding enzymes involved in biodegradation of UA, inosine and guanosine.

Gene ID	Gene	Product
ctg00001-00729	*auaG*	FAD-dependent urate hydroxylase
ctg00007-03248	*ribF*	riboflavin kinase/FMN adenylyltransferase
ctg00003-01568	*bglH*	hydroxyisourate hydrolase
ctg00005-02479, ctg00026-05485	*hpt*	hypoxanthine-guanine phosphoribosyltransferase [EC:2.4.2.8]
ctg00001-00080, ctg00032-05730, ctg00002-00970, ctg00001-00531	*rihA*, *rihB*	purine nucleosidase [EC:3.2.2.1]
ctg00013-04301, ctg00012-04009	*punA*, *deoD*	purine-nucleoside phosphorylase [EC:2.4.2.1]

## Data Availability

The original contributions presented in this study are included in the article. Further inquiries can be directed to the corresponding author.

## References

[1] Bardin T., Richette P. (2014). Definition of hyperuricemia and gouty conditions. Curr. Opin. Rheumatol..

[2] Sun L., Ni C., Zhao J., Wang G., Chen W. (2022). Probiotics, bioactive compounds and dietary patterns for the effective management of hyperuricemia: A review. Crit. Rev. Food Sci. Nutr..

[3] Ragab G., Elshahaly M., Bardin T. (2017). Gout: An old disease in new perspective—A review. J. Adv. Res..

[4] Sharma G., Dubey A., Nolkha N., Singh J.A. (2021). Hyperuricemia, urate-lowering therapy, and kidney outcomes: A systematic review and meta-analysis. Ther. Adv. Musculoskelet. Dis..

[5] Li L., Zhao M., Wang C., Zhang S., Yun C., Chen S., Cui L., Wu S., Xue H. (2021). Early onset of hyperuricemia is associated with increased cardiovascular disease and mortality risk. Clin. Res. Cardiol..

[6] Mortada I. (2017). Hyperuricemia, Type 2 Diabetes Mellitus, and Hypertension: An Emerging Association. Curr. Hypertens. Rep..

[7] Huang J., Ma Z.F., Zhang Y., Wan Z., Li Y., Zhou H., Chu A., Lee Y.Y. (2020). Geographical distribution of hyperuricemia in mainland China: A comprehensive systematic review and meta-analysis. Glob. Health Res. Policy.

[8] Chen-Xu M., Yokose C., Rai S.K., Pillinger M.H., Choi H.K. (2019). Contemporary Prevalence of Gout and Hyperuricemia in the United States and Decadal Trends: The National Health and Nutrition Examination Survey, 2007–2016. Arthritis Rheumatol..

[9] Nielsen S.M., Zobbe K., Kristensen L.E., Christensen R. (2018). Nutritional recommendations for gout: An update from clinical epidemiology. Autoimmun. Rev..

[10] Ge H., Jiang Z., Li B., Xu P., Wu H., He X., Xu W., Huang Z., Xiong T., Wang P. (2023). Dendrobium officinalis Six Nostrum Promotes Intestinal Urate Underexcretion via Regulations of Urate Transporter Proteins in Hyperuricemic Rats. Comb. Chem. High Throughput Screen..

[11] Maiuolo J., Oppedisano F., Gratteri S., Muscoli C., Mollace V. (2016). Regulation of uric acid metabolism and excretion. Int. J. Cardiol..

[12] Kakutani-Hatayama M., Kadoya M., Okazaki H., Kurajoh M., Shoji T., Koyama H., Tsutsumi Z., Moriwaki Y., Namba M., Yamamoto T. (2017). Nonpharmacological Management of Gout and Hyperuricemia: Hints for Better Lifestyle. Am. J. Lifestyle Med..

[13] Fang G., Li W., Zhang J., Ke Q., Zhu X., Long L., Li C. (2022). Safety and tolerability of available drugs for hyperuricemia: A critical review and an update on recent developments. J. Chin. Pharm. Sci..

[14] Zhao H., Lu Z., Lu Y. (2022). The potential of probiotics in the amelioration of hyperuricemia. Food Funct..

[15] Dewulf J.P., Marie S., Nassogne M.C. (2022). Disorders of purine biosynthesis metabolism. Mol. Genet. Metab..

[16] Zhang H., Xiang S., Zhai R., Li X., Hu M., Wang T., Zhang H., Pan L. (2023). Analysis of microbial and metabolic diversity in Jiangshui from Northwest China. Food Sci. Technol..

[17] Wu Y., Ye Z., Feng P., Li R., Chen X., Tian X., Han R., Kakade A., Liu P., Li X. (2021). Limosilactobacillus fermentum JL-3 isolated from “Jiangshui” ameliorates hyperuricemia by degrading uric acid. Gut Microbes.

[18] Zhao S., Feng P., Hu X., Cao W., Liu P., Han H., Jin W., Li X. (2022). Probiotic Limosilactobacillus fermentum GR-3 ameliorates human hyperuricemia via degrading and promoting excretion of uric acid. iScience.

[19] Smith D.L., Lemieux E.N., Barua S. (2018). Correction in Bicinchoninic Acid (BCA) Absorbance Assay to Analyze Protein Concentration. Nano LIFE.

[20] Du X., Jiang Y., Sun Y., Cao X., Zhang Y., Xu Q., Yan H. (2023). Biodegradation of Inosine and Guanosine by Bacillus paranthracis YD01. Int. J. Mol. Sci..

[21] Diale M.O., Kayitesi E., Serepa-Dlamini M.H. (2021). Genome In Silico and In Vitro Analysis of the Probiotic Properties of a Bacterial Endophyte, Bacillus Paranthracis Strain MHSD3. Front. Genet..

[22] Ait Seddik H., Bendali F., Cudennec B., Drider D. (2017). Anti-pathogenic and probiotic attributes of Lactobacillus salivarius and Lactobacillus plantarum strains isolated from feces of Algerian infants and adults. Res. Microbiol..

[23] Çetin B., Aktaş H. (2024). Monitoring probiotic properties and safety evaluation of antilisterial Enterococcus faecium strains with cholesterol-lowering potential from raw Cow’s milk. Food Biosci..

[24] Li T., Lyu L., Zhang Y., Dong K., Li Q., Guo X., Zhu Y. (2021). A newly isolated E. thailandicus strain d5B with exclusively antimicrobial activity against C. difficile might be a novel therapy for controlling CDI. Genomics.

[25] Brutscher L.M., Borgmeier C., Garvey S.M., Spears J.L. (2022). Preclinical Safety Assessment of Bacillus subtilis BS50 for Probiotic and Food Applications. Microorganisms.

[26] Fu X., Lyu L., Wang Y., Zhang Y., Guo X., Chen Q., Liu C. (2022). Safety assessment and probiotic characteristics of Enterococcus lactis JDM1. Microb. Pathog..

[27] Liu Y., Du J., Lai Q., Zeng R., Ye D., Xu J., Shao Z. (2017). Proposal of nine novel species of the Bacillus cereus group. nt. J. Syst. Evol. Microbiol..

[28] Fukuda D., Nolasco-Hipolito C., Gill S.R. (2021). Draft Genome Sequence of Bacillus paranthracis Strain DB-4, Isolated from Nukadoko, Fermented Rice Bran for Japanese Pickles. Microbiology Resource Announcements.

[29] Bukharin O.V., Perunova N.B., Andryuschenko S.V., Ivanova E.V., Bondarenko T.A., Chainikova I.N., Rasko D. (2019). Genome Sequence Announcement of Bacillus paranthracis Strain ICIS-279, Isolated from Human Intestine. Microbiol. Resour. Announc..

[30] Lu L., Liu T., Liu X., Wang C. (2022). Screening and identification of purine degrading Lactobacillus fermentum 9-4 from Chinese fermented rice-flour noodles. Food Sci. Hum. Wellness.

[31] Lee M.J., Khang A.R., Kang Y.H., Yun M.S., Yi D. (2022). Synergistic Interaction between Hyperuricemia and Abdominal Obesity as a Risk Factor for Metabolic Syndrome Components in Korean Population. Diabetes Metab. J..

[32] Yamada N., Saito-Iwamoto C., Nakamura M., Soeda M., Chiba Y., Kano H., Asami Y. (2017). Lactobacillus gasseri PA-3 Uses the Purines IMP, Inosine and Hypoxanthine and Reduces their Absorption in Rats. Microorganisms.

[33] Meng Y.P., Hu Y.S., Wei M., Wang K.M., Wang Y.Y., Wang S.L., Hu Q., Wei H., Zhang Z.H. (2023). Amelioration of hyperuricemia by Lactobacillus acidophilus F02 with uric acid-lowering ability via modulation of NLRP3 inflammasome and gut microbiota homeostasis. J. Funct. Foods.

[34] Kuo Y.W., Hsieh S.H., Chen J.F., Liu C.R., Chen C.W., Huang Y.F., Ho H.H. (2021). Lactobacillus reuteri TSR332 and Lactobacillus fermentum TSF331 stabilize serum uric acid levels and prevent hyperuricemia in rats. PeerJ.

[35] Li M., Wu X., Guo Z., Gao R., Ni Z., Cui H., Zong M., Van Bockstaele F., Lou W. (2023). Lactiplantibacillus plantarum enables blood urate control in mice through degradation of nucleosides in gastrointestinal tract. Microbiome.

[36] Mandal A.K., Mount D.B. (2015). The molecular physiology of uric acid homeostasis. Annu. Rev. Physiol..

[37] Chung W.-H., Kang J., Lim M.Y., Lim T.-j., Lim S., Roh S.W., Nam Y.-D. (2018). Complete Genome Sequence and Genomic Characterization of Lactobacillus acidophilus LA1 (11869BP). Front. Pharmacol..

[38] Li P., Tian W.N., Jiang Z., Liang Z.H., Wu X.Y., Du B. (2018). Genomic Characterization and Probiotic Potency of *Bacillus* sp DU-106, a Highly Effective Producer of L-Lactic Acid Isolated From Fermented Yogurt. Front. Microbiol..

[39] Liu Y., Wang S., Wang L., Lu H., Zhang T., Zeng W. (2024). Characterization of Genomic, Physiological, and Probiotic Features of Lactiplantibacillus plantarum JS21 Strain Isolated from Traditional Fermented Jiangshui. Foods.

[40] Khalil E.S., Manap M.Y., Mustafa S., Amid M., Alhelli A.M., Aljoubori A. (2018). Probiotic characteristics of exopolysaccharides-producingLactobacillusisolated from some traditional Malaysian fermented foods. CyTA J. Food.

[41] Pan M., Kumaree K.K., Shah N.P. (2017). Physiological Changes of Surface Membrane in Lactobacillus with Prebiotics. J. Food Sci..

[42] Gołaś-Prądzyńska M., Łuszczyńska M., Rola J.G. (2022). Dairy Products: A Potential Source of Multidrug-Resistant Enterococcus faecalis and Enterococcus faecium Strains. Foods.

[43] Wang X., Ying W., Dunlap K.A., Lin G., Satterfield M.C., Burghardt R.C., Wu G., Bazer F.W. (2014). Arginine Decarboxylase and Agmatinase: An Alternative Pathway for De Novo Biosynthesis of Polyamines for Development of Mammalian Conceptuses1. Biol. Reprod..

[44] Zou D., Zhao Z., Li L., Min Y., Zhang D., Ji A., Jiang C., Wei X., Wu X. (2022). A comprehensive review of spermidine: Safety, health effects, absorption and metabolism, food materials evaluation, physical and chemical processing, and bioprocessing. Compr. Rev. Food Sci. Food Saf..

[45] Cao X., Cai J., Zhang Y., Liu C., Song M., Xu Q., Liu Y., Yan H. (2023). Biodegradation of Uric Acid by Bacillus paramycoides-YC02. Microorganisms.

